# Antimicrobial Agents in Fibrous Materials: A Comprehensive Review of Natural, Inorganic, and Organic Systems

**DOI:** 10.3390/ma19142980

**Published:** 2026-07-10

**Authors:** Xueyan Que, Junqing Bai, Hai Yao, Pingping Fu, Yuanbo Xu, Xiaoyan Zhang, Yuqing Cui, Yingting Li, Jiangtao Yu, Ling Xu

**Affiliations:** 1 State Key Laboratory of Vaccines for Infectious Diseases, Xiangan Biomedicine Laboratory, State Key Laboratory of Molecular Vaccinology and Molecular Diagnostics, National Innovation Platform for Industry-Education Integration in Vaccine Research, School of Public Health, Xiamen University, Xiamen 361102, Chinam18819268303@163.com (P.F.); 15605049387@163.com (X.Z.);; 2 Yangling Hesheng Irradiation Technologies Co., Ltd., Xianyang 712000, China; baijunqing@ssnhsit.com (J.B.);; 3 Jinduicheng Molybdenum Industry Co. Ltd., Weinan 714101, China; 13772740205@163.com

**Keywords:** antimicrobial agents, natural fibers, artificial fibers

## Abstract

The escalating threat of antimicrobial resistance has spurred extensive research into antimicrobial fibers. While numerous reviews have comprehensively cataloged the classification and mechanisms of natural, inorganic, and organic antimicrobial agents, a critical gap remains: few have systematically evaluated the engineering strategies that translate intrinsic biocidal activity into durable, real-world fiber performance. This review addresses this gap by shifting focus from encyclopedic enumeration to a problem-oriented critical assessment of performance optimization strategies. We examine recent advances in natural fibers (bamboo, hemp, chitosan, jute) and synthetic fibers modified with antimicrobial agents, with emphasis on three core challenges—poor wash durability of natural agents, aggregation and leaching of inorganic nanoparticles (e.g., Ag, ZnO, MOFs), and structural limitations of organic agents (e.g., QACs, QPSs, N-halamines, PHMB). Key optimization routes, including covalent grafting, microstructural control (e.g., triaxial microfluidic spinning), organic-inorganic hybridization, and rechargeable N-halamine systems, are critically assessed for their effectiveness in enhancing washing resistance, stability, and antimicrobial synergy. Based on this comparative synthesis, we identify future directions—smart-responsive systems, sustainable processing pathways, and standardized evaluation protocols—to guide the rational design of next-generation high-performance antimicrobial fibers.

## 1. Introduction

Bacterial pathogens represent a major and persistent threat to global public health and safety. Notably, bacteria account for approximately 57% of infectious disease agents [1]. The discovery and development of well-tolerated antimicrobial drugs revolutionized medicine, enabling significant victories against bacterial infections. However, the emergence of antimicrobial resistance (AMR) is intrinsically linked to the history of antibiotic use, and the facile cross-transmission of resistance genes within populations has exacerbated this crisis [2]. Consequently, the profound benefits of antibiotics for human health are now critically threatened. This is underscored by 2019 estimates attributing 4.95 million deaths globally to AMR-associated causes, with 1.27 million deaths directly resulting from resistant bacterial infections [3]. In response, researchers are intensifying efforts to discover novel broad-spectrum antimicrobial agents or alternative strategies, such as antimicrobial peptides (AMPs) [4]. Inspired by the mechanisms underlying AMP–bacteria interactions, numerous polymeric antimicrobial materials, including hydrogels and surface coatings, have been engineered [5]. These materials demonstrate potent broad-spectrum antimicrobial activity and, by targeting bacterial biofilms, possess a lower propensity for inducing resistance compared to conventional antibiotics.

Among traditional polymeric materials, fibers hold significant prominence due to their versatility, accessibility, and extensive utilization in diverse applications, including textiles, medical devices [6,7], and household products. Derived from both natural (e.g., cotton, hemp [6], bamboo) and synthetic (e.g., polyethylene (PE), polypropylene (PP)) sources, fiber production methodologies are well-established. Growing emphasis on hygiene and infection control has driven a substantial increase in demand for effective antimicrobial fiber products. Consequently, significant research focus is directed towards developing advanced antimicrobial fibers characterized by long-lasting efficacy, broad-spectrum activity, and durable binding fastness to meet practical requirements [8].

Antimicrobial fibers are broadly categorized into natural and synthetic types. Natural antimicrobial fibers are produced by spinning inherently bioactive natural polymers, such as bamboo fiber [9,10], chitosan fiber [11,12], and hemp fiber [13]. However, these fibers often exhibit limitations, including relatively weak antimicrobial efficacy, suboptimal mechanical properties, and challenges related to dyeing and processing. To overcome these drawbacks, chemical modification or incorporation with supplementary antimicrobial agents is typically employed to enhance functionality [12,14]. In contrast, synthetic antimicrobial fibers lack intrinsic antimicrobial activity. Their antimicrobial properties are conferred through the integration of biocidal agents or via surface/structural modifications designed to elicit bactericidal or bacteriostatic effects [15].

Antimicrobial fibers achieve their functional properties primarily through the integration of antimicrobial agents, which determine the antimicrobial mechanism, efficacy, and durability. With the escalating challenge of AMR, the development of advanced antimicrobial agents and their rational loading into fibrous materials has become the key focus of research. This review aims to systematically summarize the classification, antimicrobial mechanisms of natural, inorganic, and organic antimicrobial agents, as well as their fabrication strategies and performance optimization when applied in functional fibers.

It is noteworthy that the field of antimicrobial fibers and textiles has witnessed a proliferation of review articles over the past five years, which have systematically covered the classification, mechanisms, and fabrication methods of natural, inorganic, and organic antimicrobial agents [16,17,18,19]. However, a critical gap persists: while existing reviews provide comprehensive inventories of antimicrobial agents and their basic mechanisms, few have offered a systematic and critical evaluation of performance optimization strategies—particularly from the perspective of how to achieve long-term durability, wash fastness, and synergistic enhancement in fibrous substrates. Indeed, the real challenge in translating antimicrobial agents into practical fibrous products lies not merely in their intrinsic activity but in their ability to remain functional under real-world conditions (e.g., repeated washing, mechanical abrasion, and biological environments). To address this gap, this review shifts the focus from a categorical enumeration of antimicrobial agents to a problem-oriented critical assessment of engineering strategies designed to enhance washing resistance, structural stability, and multi-target synergistic effects. We critically evaluate: (i) the limitations of natural agents (e.g., essential oils, flavonoids) and the efficacy of encapsulation, covalent immobilization, and microstructural engineering in overcoming their inherent instability; (ii) the aggregation and leaching issues of inorganic nanoparticles (e.g., Ag, ZnO, MOFs), comparing different immobilization and surface functionalization approaches; (iii) the merits and trade-offs of organic-inorganic hybrid systems in achieving synergistic antimicrobial outcomes. By distilling key insights and comparing the strengths and limitations of different optimization routes, this review aims to provide a decision-making framework for researchers and engineers developing next-generation high-performance antimicrobial fibers. Unlike prior encyclopedic summaries, this work offers a critical, comparative, and design-oriented synthesis of the field.

To ensure literature coverage, we searched Web of Science and Scopus (2000–2025) using keywords including “antimicrobial fiber”, “natural/inorganic/organic agents”, “durability”, and “wash fastness”. Inclusion criteria were peer-reviewed studies on agent-modified fibers with quantitative efficacy data; non-fiber substrates and non-English articles were excluded. From approximately 1200 initial records, 131 relevant articles were finally cited in this narrative review.

## 2. Antimicrobial Agents: An Overview

Antimicrobial agents incorporated into fibrous materials belong to a diverse class of bioactive compounds and nanomaterials. Based on their source and chemical nature, they can be classified into three core systems: natural, inorganic, and organic antimicrobial agents. These agents are the core functional components that confer bacteriostatic or bactericidal properties to fibrous materials. Their distinct chemical structures, physicochemical characteristics, and action mechanisms determine the antimicrobial efficacy, spectrum, durability, and biocompatibility of the resulting functional fibers. They are integrated into natural or synthetic fibers through fabrication strategies such as blended spinning, finishing, composite spinning, graft modification, and microstructural regulation. Their performance can be further optimized via organic-inorganic hybridization, covalent immobilization, and stimuli-responsive design to address practical challenges like poor wash resistance and limited stability.

Natural antimicrobial agents are derived from renewable biological sources (plants, animals, and microbial metabolites). They feature excellent biocompatibility, biodegradability, and low toxicity, which meet the demand for sustainable functional materials. The primary plant-derived agents include extracts from bamboo, hemp, jute, and sisal (rich in phenolic compounds, flavonoids, cannabinoids, and saponins) as well as plant essential oils (e.g., peppermint, cinnamon, and oregano oil) containing volatile aromatic active ingredients. Animal-sourced agents are represented by chitosan (a cationic polysaccharide from crustacean exoskeletons) and antimicrobial proteins from silkworm cocoons. Although natural agents are environmentally friendly, most of them have inherent limitations such as low antimicrobial potency, poor stability, and easy leaching, which reduce their long-term efficacy in fibrous materials.

Inorganic antimicrobial agents are mainly composed of metal/metal oxide nanoparticles (e.g., Ag, Cu_2_O, ZnO) and transition metal-based metal–organic frameworks (MOFs, e.g., ZIF-8, MOF-199, Ag-MOFs), along with additional inorganic nanocomposites (e.g., ZnO/Ag, ZnO/ZrO_2_/TiO_2_) engineered for synergistic performance. Their antimicrobial activity is primarily mediated by controlled metal ion release (e.g., Ag^+^, Cu^2+^, Zn^2+^) and reactive oxygen species (ROS) generation. Some metal oxides (e.g., ZnO) also exhibit photocatalytic sterilization under light exposure. Inorganic agents have high stability, broad-spectrum activity, and strong bactericidal effects, and their nanoscale morphology allows for uniform dispersion in fiber matrices. However, their practical application is restricted by challenges such as nanoparticle aggregation, potential cytotoxicity from excessive ion release, and poor binding fastness to fiber substrates (leading to reduced efficacy after repeated washing).

Organic antimicrobial agents are synthetic cationic or halogenated compounds with tunable structures, including quaternary ammonium compounds (QACs), quaternary phosphonium salts (QPSs), N-halamines, and polyguanidine/polybiguanide polymers (e.g., polyhexamethylene biguanide hydrochloride, PHMB). As the most widely used synthetic antimicrobial system for fibrous materials, their activity is typically achieved through bacterial membrane disruption (via electrostatic adsorption to anionic bacterial cell surfaces and hydrophobic alkyl chain penetration) and secondary effects such as metabolic inhibition and DNA binding. A key advantage of organic agents is the ability to adjust their antimicrobial potency, pH adaptability, and fiber compatibility by modifying structural parameters (e.g., alkyl chain length, counterion type, polymer amphiphilicity). Covalent grafting of organic agents onto fiber surfaces significantly improves wash durability, and their synergistic combination with inorganic nanoparticles (e.g., QACs-ZnO) further enhances antimicrobial performance. Notably, N-halamines exhibit unique rechargeable bactericidal properties—their antimicrobial activity can be restored by re-chlorination after depletion, making them a promising candidate for sustainable antimicrobial fibers.

For all three categories, the rational selection and combination of antimicrobial agents are guided by the application scenarios of fibrous materials (e.g., medical textiles, consumer goods, food packaging). The core design principles include balancing antimicrobial efficacy and biocompatibility, improving wash resistance and structural stability, and reducing the risk of antimicrobial resistance (AMR). The hybridization of different antimicrobial systems (e.g., natural flavonoids with inorganic nanoparticles, organic QPSs with MOFs) has emerged as a key strategy to overcome the limitations of single-agent systems, resulting in high-performance antimicrobial fibers with synergistic, broad-spectrum, and long-lasting activity.

### 2.1. Natural Antimicrobial Agents

#### 2.1.1. Bamboo Extract

Bamboo is mainly composed of structural components (such as cellulose and lignin) and nutrients (such as proteins, amino acids, sugars, vitamins, and minerals) [20]. Thompson and colleagues conducted an investigation into its natural antimicrobial properties, assessing bacterial inhibition by four raw bamboo species (*Phyllostachys Bissetii*, *Phyllostachys nigra* ‘henon’, *Phyllostachys edulis* pubescens, and *Phyllostachys rubromarginata*), natural bamboo fibers (NBFs; subjected to sterilization treatment), and commercial bamboo viscose fibers [10]. Their analysis indicated that both raw bamboo and natural bamboo fibers (NBFs) demonstrated moderate antimicrobial effects, showing greater effectiveness against *Klebsiella pneumoniae* (*K*. *pneumoniae*) than *Staphylococcus aureus* (*S*. *aureus*). Significantly, sterilized raw bamboo presented notably higher inhibition compared to unsterilized samples. For Giant Gray bamboo, the inhibition against *K*. *pneumoniae* increased from −72.97% (unsterilized) to 61.22% (sterilized), and against *S*. *aureus* from 1% to 19.9%. Similarly, sterilized Moso bamboo showed an increase in inhibition against *K*. *pneumoniae* from 40.54% to 51.84%, and against *S*. *aureus* from 1.59% to 9.14%. This improvement was ascribed to the removal of bacterial nutrients in unsterilized bamboo during the sterilization process—a phenomenon also noted in NBFs. Among the twelve commercial bamboo viscose fibers that were tested, only one exhibited bacteriostatic activity, presumably due to residual chemical additives from the processing [21]. Non-fiber elements were additionally detected within NBFs. Afrin et al. identified antimicrobial compounds in bamboo (*Phyllostachys pubescens*) through solvent extraction [22]. Although aqueous extracts were unable to inhibit *Escherichia coli* (*E*. *coli*, Gram-negative), a 20% dimethyl sulfoxide (DMSO) solution exhibited bacteriostatic (non-bactericidal) effects. Significantly, dioxane aqueous extracts attained 100% antimicrobial activity at a concentration of merely 5%, indicating that bamboo lignin contains potent, water-insoluble antimicrobial components, presumably aromatic or phenolic compounds. This conclusion was supported by Kuniyoshi et al., who identified O-Hexosyl-O-deoxyhexosyl tricin as the primary compound conferring selective antimicrobial activity against *S*. *aureus* (Gram-positive bacteria) [23]. Concurrently, stigmasterol and dihydrobrassicasterol isolated from bamboo shoot bark demonstrated broader-spectrum activity against both *S*. *aureus* and *E*. *coli* [24].

**Figure 1:**
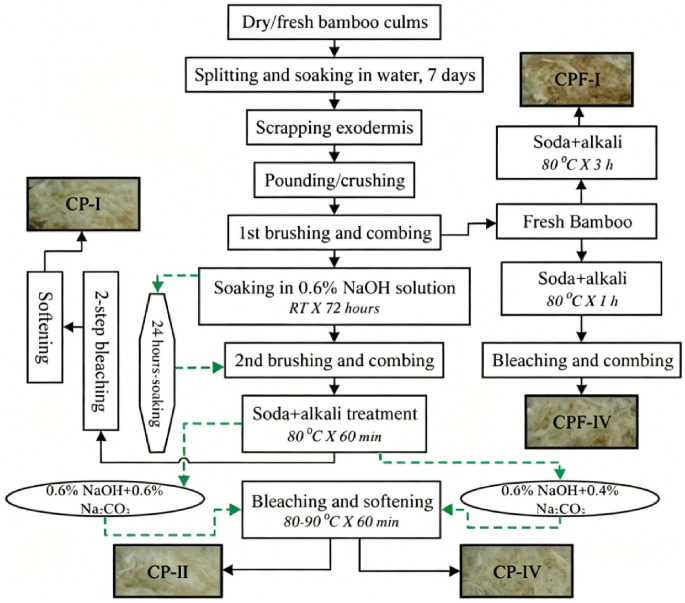
Extraction process of natural bamboo fiber, including initial treatment, enzymatic and chemical delignification, modification, and softening for various specimens [9].

Thompson and colleagues further investigated the spinnability and antimicrobial properties of *Phyllostachys rubromarginata* fibers that had been processed through six different methods, as shown in Figure 1 [9]. All the resulting NBFs demonstrated antimicrobial effects against *S*. *aureus*. Among them, CPE-XI fibers were produced through combined mechanical, chemical, and various enzymatic treatments. Mechanical treatments encompass crushing in a milling machine, brushing, carding with a steel hand-operated brush, agitation, and boiling. Chemical treatments involve either pretreatment or posttreatment using NaOH, NaHCO_3_, and H_2_O_2_, as depicted in Figure 1. It showed superior antimicrobial activity, as evidenced by larger inhibition zones. Enzymatic treatment retained a higher proportion of intrinsic antimicrobial components while causing less delignification, although it led to poorer spinnability. In contrast, CP-I fibers, obtained via chemical delignification, had enhanced yarn-making qualities, being finer and softer, and still maintained considerable, albeit slightly reduced, antimicrobial activity compared to CPE-XI. Notably, both processed fiber types (CP-I and CPE-XI) outperformed raw bamboo in terms of antimicrobial efficacy, indicating that appropriate thermal or mild chemical treatments can effectively enhance the fibers’ inherent antimicrobial properties [25]. The study further demonstrated that extraction using a 1.5% NaOH solution maximized the enhancement of antimicrobial activity, whereas higher concentrations of NaOH diminished this effect. This is probably due to the concurrent sterilizing effects of increased temperatures and the elimination of water-soluble nutrients that would otherwise facilitate bacterial proliferation.

The natural antibacterial mechanism of bamboo extract is a complex process involving multiple targets and pathways. It mainly exerts a synergistic effect through various methods, such as damaging microbial cell structures, interfering with energy metabolism, inducing oxidative stress, and inhibiting biofilm formation, ultimately resulting in microbial death.

#### 2.1.2. Hemp Extract

Industrial hemp, scientifically classified as Cannabis sativa L. and characterized by a low tetrahydrocannabinol (THC) content (<less than 0.3%), is mainly cultivated for the production of fiber, seeds, and oil, thereby differentiating it from medicinal varieties [26,27]. Researchers have extracted bioactive compounds from hemp through the use of organic solvents, such as ethanol, hexane, and hexanoic acid. Extracts obtained from C. sativa exhibit broad-spectrum antimicrobial activity against clinically significant pathogens, including *S*. *aureus*, *E*. *coli*, *Pseudomonas aeruginosa* (*P*. *aeruginosa*), *Acinetobacter baumannii* (*A*. *baumannii*), and *Stenotrophomonas maltophilia* (*S*. *maltophilia*) [28,29,30]. Likewise, hemp essential oils demonstrate notable antimicrobial effects against various strains of *S*. *aureus* with different antibiotic susceptibility profiles [31]. Furthermore, hemp seed oil, in conjunction with ethanolic and hexanoic seed marc extracts, exhibits inhibitory activity against Gram-positive bacteria, including *S*. *aureus* and *Cutibacterium acnes* (*C*. *acnes*) [32]. Crucially, these antimicrobial properties are applicable to multiple antibiotic-resistant clinical isolates, particularly methicillin-resistant *S*. *aureus* (MRSA) [28,32,33]. and multi-drug-resistant *K*. *pneumoniae* [29]. The observed antimicrobial effects are primarily attributed to cannabinoids and their derivatives [34,35,36,37,38], or secondary metabolites such as flavonoids, phenols, and phenolic acids [31], which disrupt bacterial membrane integrity and biofilm formation [39].

**Figure 2:**
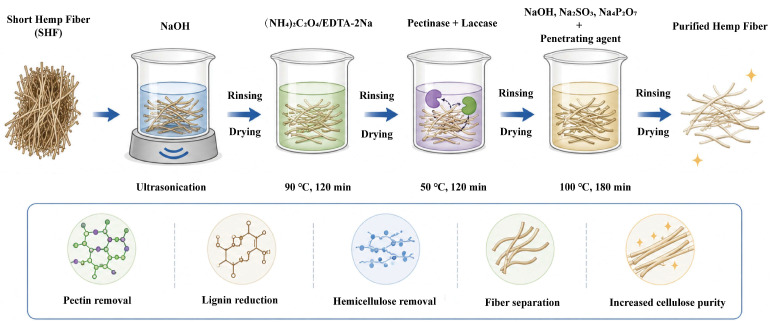
The impurity removal process for seed-type hemp fibers using ammonium oxalate–complex enzyme–chemical and EDTA-2Na–complex enzyme–chemical methods, including ultrasonic treatment, chelation, enzymatic reaction, and chemical washing (recreated according to reference [40]).

In the pursuit of functional materials, Zhao and colleagues successfully converted seed-type hemp into regenerated cellulosic fibers through sequential degumming, pulping, and spinning processes [40] as depicted in Figure 2. The resultant fibers exhibited significant antimicrobial effectiveness, with a 76.63% inhibition rate against *E*. *coli* and a 46.35% inhibition rate against *S*. *aureus*. In the context of translating this potential into consumer-oriented applications, South Korean textile innovators have recently developed antimicrobial socks. They achieved this by integrating lyocell fibers with hemp stem bark extract fibers through advanced knitting technology [41]. These products attained remarkable efficacy, exhibiting 99.9% inhibition of bacterial growth and the formation of associated odors during perspiration, while substantially improving foot health indicators. Consumer approval was manifested in a satisfaction rating of 4.63 out of 5 points.

Cannabis extract exerts antibacterial effects through multiple mechanisms, which are as follows: (1) Electrostatic binding to anionic phospholipids results in an increase in membrane permeability and the leakage of cytoplasm and ions, thereby leading to the death of Gram-positive bacteria. (2) It interferes with energy metabolism (ATP synthesis and the TCA cycle); CBD reduces ATP and enzyme activity, exacerbating metabolic disorders. (3) It induces the accumulation of reactive oxygen species (ROS) and oxidative stress; gene up-regulation initiates the ROS cascade, which damages membrane integrity through lipid peroxidation. (4) It inhibits quorum sensing; CBD interferes with signaling, reduces cell hydrophobicity and adhesion, and suppresses biofilm formation.

#### 2.1.3. Chitosan Extract

Chitosan, a cationic polysaccharide from crustacean shells, carries primary amines that protonate in acidic media, conferring antibacterial activity [11,42,43,44,45,46]. When processed into fibers via wet spinning or electrospinning, its efficacy depends on molecular weight (Mw) and degree of deacetylation (DD) [11,47,48,49]. For samples with comparable DD values, the inhibitory rate of chitosan fiber against *S*. *aureus* initially increases and subsequently decreases with an increasing Mw, whereas their bactericidal activity against *E*. *coli* decreases as Mw increases. Even though the chitosan fibers are from different manufacturers, the results remain the same. The optimal combination of Mw and DD can be selected to attain the targeted antibacterial activity of chitosan fibers [11,50].

**Figure 3:**
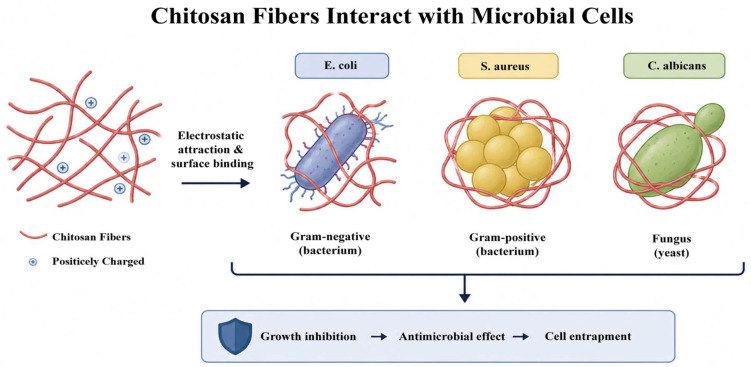
Interaction between chitosan fibers and bacterial cells. Chitosan fibers show strong activity against Gram-positive/negative bacteria and fungi. For bacteria (e.g., *S*. *aureus*, *E*. *coli*), the mechanism involves surface adsorption and electrostatic interaction: protonated chitosan binds to negatively charged cell surfaces, blocks nutrients, and disrupts membrane permeability. For fungi (e.g., *C. albicans*), the action is more complex: it disrupts the membrane, chelates trace elements to starve cells, and penetrates to interfere with DNA/protein synthesis (adapted and redrawn from [11]).

As shown in Figure 3, the principal antimicrobial mechanism entails an electrostatic interaction between the protonated amine groups on chitosan fibers and the anionic components on the bacterial cell surface. This disrupts the integrity of the biofilm, enhances the membrane permeability, and leads to the leakage of intracellular constituents, ultimately resulting in cell death [50,51]. Antifungal action is carried out through three sequential mechanisms: (1) Electrostatic binding to anionic phospholipids enhances membrane permeability, leading to cytoplasmic leakage; (2) Chelation of essential trace metals disrupts the acquisition of nutrients; (3) Penetration of the cell wall followed by DNA binding impairs the synthesis of critical proteins and enzymes [11,50,52].

#### 2.1.4. Jute Extract

**Figure 4:**
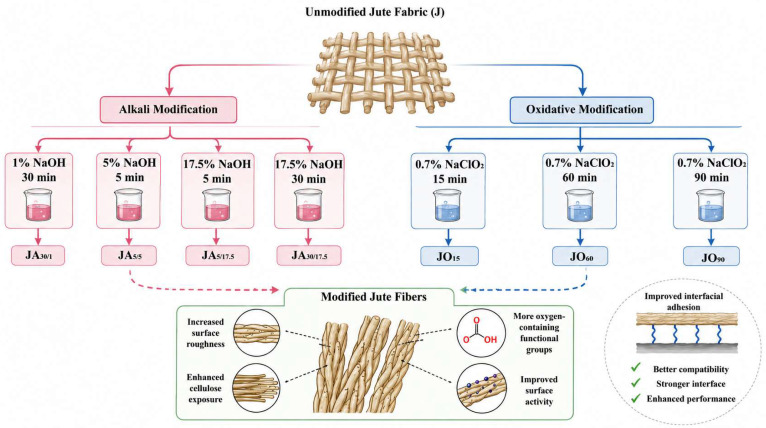
Scheme of jute fabric chemical modifications and sample codes. Alkali treatments with NaOH (1% for 30 min, 5% for 5 min, 5% and 17.5% for 30 min) produce samples JA_30/1_, JA_5/5_, JA_5/17.5_, JA_30/17.5_. Oxidative treatments with 0.7% NaClO_2_ for 15, 60, and 90 min yield JO_15_, JO_60_, and JO_90_. These modifications selectively remove hemicelluloses or lignin (recreated from reference [56]).

Jute (*Corchorus capsularis* L. [*C. capsularis*] and *Corchorus olitorius* L. [*C. olitorius*]), a bast fiber crop belonging to the Malvaceae family, provides sustainable material solutions for food packaging applications on account of its inherent antimicrobial properties [53]. Ethanol extracts of *C. olitorius* leaves exhibit significantly larger antimicrobial inhibition zones compared to ethanol/water or aqueous extracts. This can be ascribed to ethanol’s superior extraction efficiency for bioactive phenolic acids and flavonoids [54]. Nonetheless, divergent findings are present: certain studies report the absence of detectable antimicrobial activity in ethanolic extracts of *C. olitorius* leaves, possibly attributable to sub-threshold concentrations of active constituents [55]. Figure 4 presents the different chemical modification schemes (alkali and oxidative treatments) used to obtain multifunctional jute fabrics with various sample codes (e.g., JA30/1, JA5/5, JA30/17.5, JO15, JO60, JO90) [56]. In separate antibacterial tests, commercial jute fabrics containing 60.09% cellulose achieved significant bacterial reduction rates of 99.11% against *E*. *coli* and 99.28% against *S*. *aureus* within a 24 h period. Overall, these findings validate the substantial intrinsic antimicrobial potential of jute for use in functional packaging materials.

The natural antibacterial mechanism of jute originates from the synergy between its bioactive components and distinctive physical structures, which involves bacteriostasis based on chemical and physical structures. Chemically, the hydrophobic components in jute, such as flavonoids and phenols, penetrate bacterial cell membranes, interact with the phospholipid bilayer to disrupt membrane integrity and increase permeability. This leads to the leakage of intracellular contents, such as K^+^, ATP, and proteins, and ultimately causes cell death. Moreover, jute extract interferes with bacterial energy metabolism by inhibiting ATP synthesis and disrupting the tricarboxylic acid cycle and oxidative phosphorylation. This significantly reduces intracellular ATP levels and the activities of ATPase and alkaline phosphatase, thereby exacerbating energy metabolism disorders and inhibiting bacterial proliferation. Physically, the unique porous cavity structure of jute fibers stores abundant oxygen to form an aerobic micro-environment that inhibits the survival of anaerobic bacteria. The rough surface texture and micropores reduce the bacterial adhesion area, and the synergy between multi-scale and oxygen-storing cavity structures enhances antibacterial efficacy.

#### 2.1.5. Essential Oil

Plant-derived antimicrobial compounds, including flavonoids and polyphenols, are widely used in medicine, consumer goods, and food industries due to their broad-spectrum efficacy and biocompatibility. Essential oils, which are volatile aromatic extracts from species-specific plant organs such as flowers or leaves, exhibit significant antimicrobial activity. Key examples include peppermint, cinnamon, and lemongrass [57,58,59], rosemary, and oregano [60]. These oils can be incorporated into antimicrobial fibers via electrospinning, where a co-solution of essential oil and polymer (e.g., poly (ε-caprolactone), PCL) undergoes jetting under high-voltage electrostatic fields [58,60].

In peppermint oil, bioactive constituents (menthol, menthone, piperitenone oxide, carvone) drive antimicrobial effects [57,61]. Boccaccini et al. electrospun peppermint oil/PCL fibers dissolved in glacial acetic acid. [58] Although the incorporation of oil led to a slight reduction in fiber diameters, it preserved a homogeneous morphology without any aggregation. A higher oil loading (6% *v*/*v*) improved the antimicrobial efficacy, decreasing the viability to 50 ± 3% (for *S. aureus*) and 70 ± 2% (for *E. coli*) after 24 h, which was significantly lower than that of the pure PCL controls (80% and >100%, respectively). Meanwhile, it demonstrated negligible cytotoxicity towards normal human dermal fibroblasts. This selective toxicity stems from the structural disparities in microbial membranes: the lipopolysaccharide barrier of *E. coli* hinders the penetration of the compound, which accounts for its lower susceptibility compared to the Gram-positive *S. aureus* [62,63]. Similar trends occur in cinnamon oil/PVA fibers. Notably, *S. aureus* exhibits growing resistance to conventional antibiotics and plant agents, yet cinnamon oil retains superior efficacy against this pathogen [64,65].

**Figure 5:**
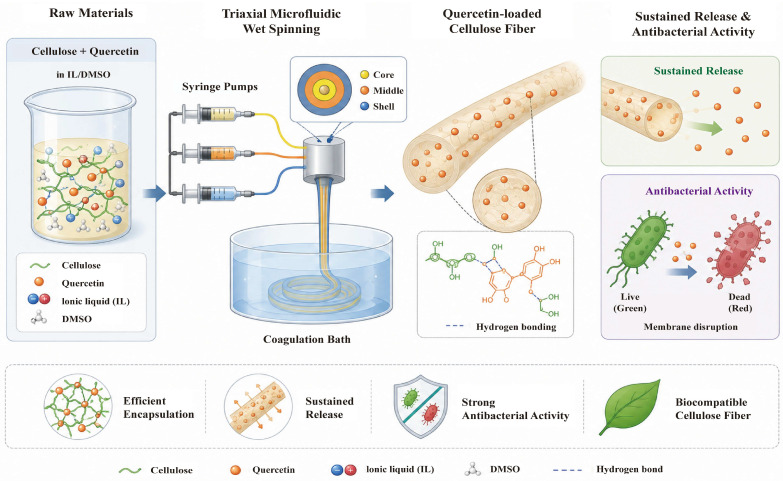
The Triaxial Microfluidic Spinning process for preparing antibacterial quercetin/cellulose fibers (Q-RCFs). The inner and outer channels contain IL/DMSO/cellulose spinning dope, while the middle channel contains quercetin/IL/DMSO/cellulose dope. The three coaxial flows merge and enter a water coagulation bath, where solvent exchange occurs, forming fibers with controlled quercetin distribution for enhanced antibacterial activity (recreated from reference [69]).

Wash durability is of crucial importance for practical applications. Thymol/cellulose fibers produced through ionic liquid-assisted dry-jet wet spinning (with 25% thymol) demonstrated an inhibition rate of over 90% against *E. coli* and *S. aureus*. After 15 washes, these fibers retained 75% of their activity, outperforming conventional finishing methods [66,67]. Phenolic compounds like thymol disrupt cell membranes, altering transmembrane potential and inducing adenosine triphosphate (ATP) efflux [68]. Microstructural control further improves durability. Nie et al. fabricated quercetin-incorporated cellulose fibers via triaxial microfluidic spinning with 1-Ethyl-3-methylimidazolium diethyl phosphat ([Emim]DEP) ionic liquid (IL) as shown in Figure 5 [69]. By adjusting the inner, intermediate, and outer flow rates (IL/DMSO/cellulose solution), the spatial distribution of quercetin was controlled. When the flow rates were 0/15/0 mL/h, discrete patterning was achieved, with an anti-*S. aureus* efficacy of 84.9%. When the flow rates were 5/2.5/7.5 mL/h, outward-diffused dense packing was obtained, with an efficacy of 93.3%. After 30 washes using two methods, the efficacy remained above 84.6% due to the reservoir-driven replenishment of quercetin.

Conversely, the microencapsulation of Artemisia annua essential oil in Polymethyl Methacrylate (PMMA) through wet spinning produced composite fibers with inhibition rates of 89.8% against *E. coli* and 97.8% against *S. aureus*, although the mechanical properties were impaired [67,70,71]. Activity decreased by only 4.5–5.2% after 30 washes, demonstrating competitive wash fastness.

The covalent immobilization of antimicrobial agents on fiber surfaces offers a reliable strategy for attaining long-lasting antimicrobial functionality. Ferrero et al. exemplified this approach by means of UV-initiated grafting of chitosan onto cotton fabrics. In this process, optimized process parameters ensured consistent antimicrobial performance even after multiple washing cycles, and the efficacy was further verified in semi-industrial scale-up trials [72]. Employing a complementary chemical approach, Cao accomplished the cross-linking immobilization of chitosan oligosaccharide onto cellulose fibers, utilizing citric acid as the cross-linker and sodium hypophosphite as the catalyst [73]. The resultant fibers, with a chitosan oligosaccharide loading of 61.77 mg/g, demonstrated complete inhibition (100%) against both *S. aureus* and *E. coli* after 12 h of exposure, along with a 99.19% reduction in MS2 bacteriophage within one hour. Wash durability testing indicated remarkable persistence against *S. aureus* (99.99% inhibition after 30 cycles), whereas the inhibition of *E. coli* showed a gradual decrease (98.41% after 20 cycles) as a result of ester bond hydrolysis at the grafting sites. Significantly, the antiviral efficacy against MS2 phage remained highly effective (98.38% after 20 washing cycles), validating the stability of the covalent immobilization approach across microbial targets.

#### 2.1.6. Other Natural Antimicrobial Agents

**Figure 6:**
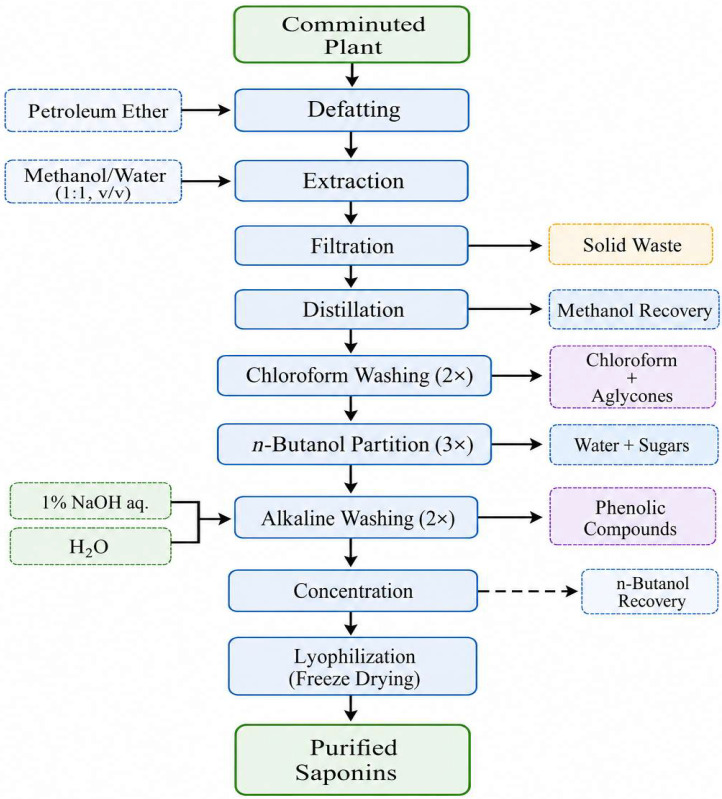
Preparation of saponin-rich extracts from sisal and juá. Dried, comminuted. Sieved plant material is first defatted with petroleum ether in a Soxhlet extractor. The residue is then extracted with methanol/water (1:1). After methanol removal and filtration, the aqueous phase is washed with chloroform, followed by butanol partition. The butanolic phase is subsequently washed with 1% NaOH to remove phenolic glycosides and then neutralized with water. Butanol is removed by rotary evaporation, and the saponins are suspended in water and lyophilized, yielding the purified saponin extract (recreated from reference [74]).

Natural fibers possessing inherent antimicrobial properties encompass plant-derived types, such as sisal (Agave sisalana). The conditions were a pH ranging from 3 to 4 at near-room temperature, or a pH within the range of 10–11 at temperatures around 55–60 °C. The preparation of a saponin-rich extract is presented in Figure 6 [74]. Furthermore, regarding coir (derived from the husks of Cocos nucifera), the minimum inhibitory concentrations (MICs) of the extract were within the range of 0.39 to 12.50 mg/mL. The time-kill assay indicated that, following a 15 min contact time with the fractions at 1 × MIC, a minimum of 27.8% of the target was killed, and after 120 min, a minimum of 95% was killed [75]. In addition to animal-sourced fibers such as silkworm cocoons, they contain numerous antimicrobial proteins, among which protease inhibitors and seroins are the most abundant components. Protease inhibitors exhibit antifungal activities by binding to fungal Pathogen-Associated Molecular Patterns (PAMPs) and/or inhibiting fungal proteases, while seroins play antibacterial, antifungal, and antiviral roles [76].

#### 2.1.7. Comparison of Nature Antimicrobial Agents

The comparative summary in Table 1 highlights that the antimicrobial performance of natural agents is not an intrinsic constant but a complex function of chemical structure, extraction/processing conditions, and the target bacterial environment. Why some systems perform better: For example, hemp and chitosan show broad-spectrum activity, but chitosan’s strong electrostatic killing (via –NH_3_^+^) is superior under acidic conditions (e.g., wound fluid), whereas hemp’s multi-component membrane integration is more effective against drug-resistant strains like MRSA even at neutral pH. How chemical composition influences efficacy: The presence of hydrophobic phenolics in bamboo and jute favors interaction with Gram-negative outer membranes, while the cationic nature of chitosan directly targets the anionic teichoic acids of Gram-positive bacteria. How processing modifies activity: Alkali treatment of bamboo can enhance efficacy by removing competing nutrients, but over-delignification destroys active lignin-bound flavonoids; similarly, encapsulation or ionic-liquid spinning is essential for essential oils to survive high-temperature fiber manufacturing. Which mechanisms dominate under realistic conditions? In a dry, short-contact application (e.g., face mask), the rapid membrane disruption by chitosan or essential oils is desirable. In a hydrated, long-term wound dressing, the sustained.

### 2.2. Artificial Antimicrobial Agents

#### 2.2.1. Inorganic Agents

Inorganic antimicrobial agents, mainly composed of metals, metal oxides, and their nanoparticle derivatives, achieve antimicrobial effects through properties such as uniform size distribution, spherical morphology, positive surface charge, and hydrophobicity. Zhu et al. fabricated polyethylene terephthalate (PET) composite fibers through in situ polymerization with cuprous oxide (Cu_2_O) immobilized on zirconium phosphate (ZrP) nanosheets [77]. This approach averted the aggregation of Cu_2_O, which is a common phenomenon in conventional melt spinning, resulting in superior nanoparticle dispersion and improved mechanical properties. At a mere 0.2 wt% loading, nano-Cu_2_O@ZrP/PET fibers attained over 99% inhibition against both *E. coli* and *S. aureus*. Notably, these composites exhibited enhanced effectiveness against *C. albicans* (92% inhibition at 0.2 wt% Cu_2_O) when compared to PET/Cu_2_O controls (85%). Remarkably, the effectiveness against *C. albicans* remained at over 99% inhibition even with an ultralow 0.07 wt% Cu loading. Alternatively, micro-dissolved cotton fabrics functionalized via Cu^2+^ immersion and high-temperature polydimethylsiloxane reduction achieved uniform, non-aggregated Cu_2_O surface deposition [78]. These fabrics demonstrated broad-spectrum activity, with inhibition zones measuring 10 mm for *Bacillus subtilis* (*B. subtilis*), 3 mm for *S. aureus,* 2 mm for *P. aeruginosa*, and 2 mm for *E. coli.* Nevertheless, limitations in wash durability became apparent as the zones decreased to 4 mm, 2 mm, 1.5 mm, and 0.3 mm, respectively, after 20 washing cycles, suggesting poor binding fastness. The antimicrobial mechanism of copper consists of sequential stages [79]:

The mechanism of action of copper-based antimicrobial agents entails a continuous process. Initially, electrostatic adsorption takes place between cuprous oxide particles and the surface of microorganisms. Subsequently, a dual-pathway damage is established through the slow release of copper ions and the generation of reactive oxygen species. Copper ions disrupt the structure of biological molecules and inactivate proteins, whereas reactive oxygen species induce oxidative stress in microorganisms, lead to membrane leakage, and trigger inflammatory responses, ultimately initiating the cell death mechanism of microorganisms [79,80,81].

The oxidative stress induced during this process inflicts damage on bacteria when certain Cu_2_O particles penetrate the cells, ultimately leading to structural collapse manifested as cell wall deformation and cytoplasmic membrane rupture. This catastrophic breakdown results in extensive leakage of the cytoplasm, thereby accelerating the death of bacterial cells [82].

Silver nanoparticles (AgNPs) are typically synthesized by reducing Ag^+^ ions on fiber surfaces using NaBH_4_, or bioreducing agents like tea polyphenols [83,84,85]. Their antibacterial action relies on sustained Ag^+^ release, which alters membrane permeability and induces intracellular ROS accumulation [86]. The efficacy scales with Ag^+^ concentration, fiber length, and silver loading [87].

A major drawback is poor wash fastness due to physical deposition. To address this, several immobilization strategies have been developed: (i) chelation via hydroxyl groups of binders; (ii) mussel-inspired polydopamine/polyethyleneimine coatings; (iii) surface cationization through carboxyl-enrichment (e.g., selective oxidation followed by butanetetracarboxylic acid grafting) [88,89]. The last approach is most effective: cotton fibers maintained 100% inhibition against *S*. *aureus* and *E*. *coli* after 80 washes; polyacrylic acid adsorption gave 97.9–99.0% retention under the same conditions [90,91]. These results confirm that carboxyl-rich surfaces strongly anchor AgNPs, enabling durable antimicrobial textiles.

Zinc oxide (ZnO) is a widely used antimicrobial agent, which is highly regarded for its non-toxicity, cost-effectiveness, stability, and durability. However, it shows moderate antimicrobial efficacy against both Gram-negative and Gram-positive bacteria. Comparative studies of melt-spun fibers modified with either ZnO or chitosan revealed that at a 3% ZnO loading, there was 88.23% bacterial inhibition, whereas at 0.5 wt% chitosan, a 97% reduction was achieved [92]. To enhance efficacy, researchers have combined zinc oxide (ZnO) with various metals and metal oxides. Specifically, they have developed heterostructured zinc oxide/silver (ZnO/Ag) composites, blended these composites with polylactic acid, and then dip-coated the resulting mixture onto polyethylene (PE) fibers [93]. The resultant antimicrobial polyethylene (PE) fibers demonstrated outstanding mechanical properties and wear resistance. Simultaneously, they achieved an inhibition rate of over 99.9% against *E*. *coli* and Bacillus subtilis, and retained efficacies of 99.2% and 99.6%, respectively, after 50 washing cycles. Research indicates that zinc-based nanohybrid materials exert antimicrobial effects via dual mechanisms, namely metal ion release and photocatalytic activity [93,94,95,96]. Upon coming into contact with the fiber surface, bacteria interact with the liberated Zn^2+^ and Ag^+^ ions. These ions bind to and deactivate essential bacterial proteases, ultimately leading to cell death. Moreover, when exposed to sunlight (especially UV light), ZnO nanoparticles undergo aqueous and atmospheric photoactivation, generating electron–hole pairs. These electron–hole pairs produce reactive oxygen species that can oxidize microorganisms, thereby achieving efficient sterilization. ZnO-based composites that incorporate metal oxides such as CuO, Fe_2_O_3_, and ZrO_2_/TiO_2_ display significant antimicrobial efficacy. Among them, ZnO/ZrO_2_/TiO_2_ nanocomposites exhibit particularly remarkable photocatalytic activity [97,98,99]. Similarly, heterogeneous systems combining ZnO with non-metallic components such as chitosan, graphene, silica, and alginate display robust antimicrobial performance [100,101,102,103].

**Figure 7:**
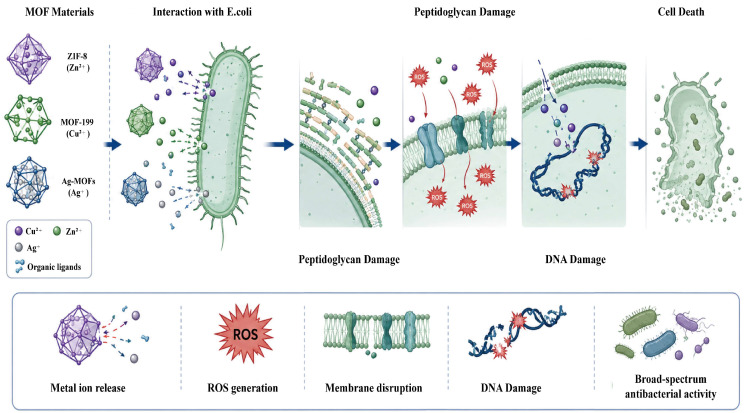
Schematic diagram of the bactericidal mechanism for MOF of different metals (ZIF-8, MOF-199, and Ag-MOFs) against *E*. *coli.* Metal ions (Zn^2+^, Cu^2+^, Ag^+^) are released from the MOFs, which attach to the bacterial cell membrane, penetrate it, and cause membrane damage. This leads to cell lysis, protein inactivation, and DNA fragmentation, ultimately resulting in bacterial death (recreated from reference [104]).

Transition metal-based metal–organic frameworks (MOFs) exhibit antimicrobial activity by means of controlled metal ion release. Yang et al. fabricated cellulose composite fibers—ZIF-8@CF, MOF-199@CF, and Ag-MOFs@CF—through the in situ deposition of zinc-, copper-, and silver-based MOF crystals, respectively [104]. As depicted in Figure 7, these MOF-functionalized fibers release metal ions and organic ligands, which induce bacterial membrane disruption and DNA fragmentation, thereby demonstrating exceptional antimicrobial performance. Against *E. coli*, the measured inhibition zone diameters were 9.1 mm (ZIF-8@CF), 15.2 mm (MOF-199@CF), and 20.8 mm (Ag-MOFs@CF). When utilized in air filtration systems, these materials retained effective gas adsorption and aerosol particle filtration capabilities. Additionally, research indicates that wet-spun ZIF-8-modified alginate fibers achieved 100% antimicrobial efficacy with a crystal loading of merely 0.4%, while alginate fibers functionalized with ZIF-67 (a cobalt-based MOF) exhibited complete (100%) antimicrobial efficacy at particle loadings exceeding 0.15 wt% [105,106].

#### 2.2.2. Organic Agents

Cationic synthetic antimicrobial polymers incorporating quaternary ammonium, amine, and phosphonium groups have attracted significant interest due to their synthetic versatility and broad-spectrum antimicrobial efficacy. The antimicrobial activity and physicochemical properties of these polymers can be tailored by modulating key structural parameters, including monomeric composition, counterion selection for charged groups, polymer amphiphilicity, and alkyl chain length on cationic centers.

**Figure 8:**
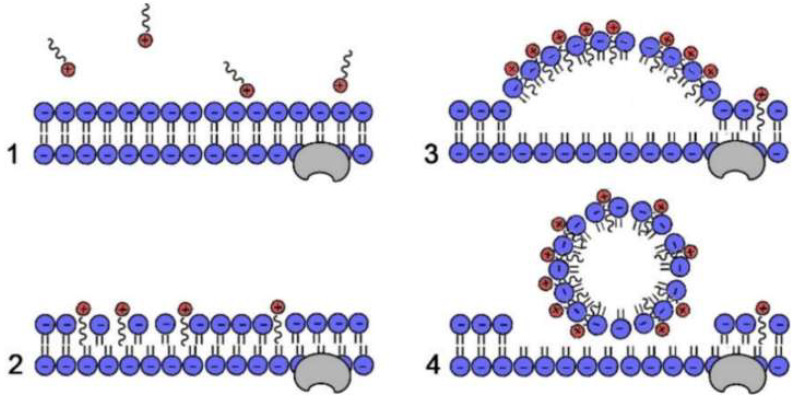
The antimicrobial mechanism diagram of QACs: where the phospholipid membranes are depicted in blue and the QACs are shown in red. (**1**) Adherence to the bacteria’s surface through electrostatic forces; (**2**) Penetration of the cell membrane and attachment to it; (**3**) Reaction with cytoplasmic membrane to cause disorder; and (**4**) Membrane damage [108].

Quaternary ammonium compounds (QACs), characterized by at least one positively charged nitrogen atom (derived from aromatic rings like imidazole, quinoline, isoquinoline, or pyridine, or from linear chains) with typically Cl^−^ or Br^−^ counterions, represent the most prevalent class of organic antimicrobial agents [107,108], as shown in Figure 8. Their bactericidal mechanism involves four sequential steps: (1) electrostatic adsorption to bacterial cell walls, (2) hydrophobic alkyl chain penetration of lipid bilayers, causing structural destabilization and increased permeability, (3) disruption of membrane ionic equilibrium, and (4) cytoplasmic leakage leading to cell death [108]. When grafted onto cotton fibers via a two-step oxidation-grafting process, these functionalized fibers demonstrated >99% efficacy against *E. coli*, *S. aureus*, and *C. albicans* [109]. Undoubtedly, grafting provides superior wash durability compared to physical coating. Song et al. confirmed this advantage by grafting diallyldimethylammonium chloride onto polypropylene (PP) fiber membranes [110]. While both plasma-treated grafted and physically coated samples initially achieved ~100% *E. coli* eradication, the grafted samples maintained 99.99% bactericidal activity after 12 min of ultrasonic cleaning versus 64.33% for coated samples. After 30 min, air-, Ar-, and Ar/O_2_ plasma-treated samples retained 91.67%, 87.97%, and 73.95% efficacy, respectively, whereas coated samples lost all activity. The antimicrobial performance can be further enhanced through QACs-ZnO synergism or by combining photodynamic therapy with QACs contact killing, significantly boosting fiber sterilization capacity [111,112,113].

Quaternary phosphonium salts (QPSs) constitute another significant class of cationic antimicrobial agents succeeding quaternary ammonium compounds (QACs). Belonging to the same group V element as nitrogen but possessing a larger atomic radius, the phosphorus atom exhibits enhanced polarization, which promotes adsorption onto negatively charged bacterial surfaces. Its lower electronegativity also facilitates a more uniform distribution of cationic charge, further augmenting bactericidal efficacy. Liu et al. systematically developed polyacrylonitrile composite fibers functionalized with QPSs bearing alkyl chains of varying lengths (C1, C2, C6, C8, C12) [114]. Their study demonstrated that longer alkyl chains (C12) conferred superior binding stability within the fiber matrix and enhanced antimicrobial performance. Specifically, the C12-modified fibers achieved complete eradication (100% efficiency) of *E. coli*, *S. aureus*, *P. aeruginosa*, and *C. albicans* within 2 h. Remarkably, after 50 washing cycles, these fibers retained high efficacy levels of 95.33%, 96.67%, 93.84%, and 92.11% against the respective pathogens. Furthermore, QPSs with longer alkyl chains (exceeding C6) exhibited potent pH-independent antimicrobial activity, achieving >99% efficacy across the tested pH range (3–10). In contrast, those with shorter chains (shorter than C6) displayed pH-dependent activity, with efficacy dropping significantly (~70%) at pH 5.5. The antimicrobial mechanism involves a synergistic combination of electrostatic interactions and hydrophobic effects, wherein the hydrophobic alkyl chains disrupt the integrity of the cytoplasmic membrane [115].

Haloamines, which are compounds characterized by nitrogen-bound halogen groups, are predominantly exemplified by chloramines. Structurally, they are classified as amides, imides, or amines. Sun et al. devised a sequential “pad-dry-cure” treatment to graft 3-allyl-5,5-dimethylhydantoin onto high-performance fibers, including poly(m-toluene dicarboxamide) (Nomex), poly(aromatic imide) (Kermel), and poly(benzimidazole)/poly(terephthalamide) (Kevlar). When exposed to chlorine gas, hydantoin groups are converted into active chloramine structures [116]. The modified fibers attained over 99% antimicrobial efficacy against both *E. coli* and *S. aureus* following 120 min of contact. Although the active chlorine content decreased significantly after 50 washing cycles, re-chlorination completely restored the original antimicrobial performance.

Polyguanidine and polybiguanide polymers display remarkable water solubility and broad-spectrum antimicrobial activity. Polyhexamethylene biguanide hydrochloride (PHMB), a representative guanidine-based polymer, is synthesized through the polycondensation of hexamethylene diamine chloride and dicyandiamide [117]. Tan et al. fabricated antimicrobial fibers by covalently grafting polyhexamethylene biguanide (PHMB) onto polymeric substrates that were prepared via the polymerization-wet spinning of styrene and glycerol methacrylate. This approach resulted in a 100% inhibition rate against both *E*. *coli* and *S*. *aureus*, and the fibers maintained full efficacy after 50 washing cycles [118]. These guanidine-based polymers achieve their antimicrobial effects via multiple synergistic mechanisms, namely metabolic inhibition, cell membrane disruption, and DNA-binding-mediated transcriptional interference, which jointly reduce the adaptation potential of bacteria [119]. Furthermore, their antimicrobial performance can be enhanced through either combination with inorganic particles (e.g., ZnO, Ag) or topological structural optimization of the polymer itself [120,121,122].

### 2.3. From Antimicrobial Agents to Antimicrobial Fibers

Generally, antimicrobial fibers are fabricated by integrating active agents through blending, composite spinning, surface grafting, or finishing treatments [123,124]. Each method differs significantly in antimicrobial agent incorporation, washing durability, and environmental impact.

Melt blending involves physically mixing antimicrobial agents with polymer resins prior to spinning and embedding agents with the fiber matrix. This method achieves moderate wash durability, as external agents are gradually lost while internally embedded agents remain available. Wendler et al. [125] demonstrated that copper-loaded Lyocell fibers retained strong antibacterial activity after 50 household washes. However, the homogenous dispersion of inorganic nanoparticles in polymer fibers remains challenging. For ecotoxicity, melt blending substantially reduces environmental release because embedded agents are less prone to leaching during laundering, thereby mitigating aquatic contamination.

Graft modification covalently bonds antimicrobial groups to fiber macromolecules, offering superior wash durability. Li et al. grafted dodecyl(triphenyl)phosphonium bromide onto bamboo cellulose molecules; after 50 washing cycles, the antimicrobial agent retained stable bonding [126]. Similarly, Zhu et al. reported phytic acid-halamine grafted cotton fibers maintained self-extinguishing and antibacterial properties after 50 washes, with antibacterial efficacy fully restorable through simple bleaching treatment [126]. Due to covalent bonding, grafted agents exhibit negligible leaching, representing the most ecologically benign approach.

Bicomponent spinning incorporates antimicrobial agents within the sheath layer of sheath-core fibers, requiring less additive while achieving good durability. This method provides moderate environmental risk, as agents are partially protected but may still be released from sheath surfaces over extended use.

Finishing treatment applies antimicrobial agents via dipping or coating, offering simple operation but poor wash durability. Agents are only physically adsorbed onto fiber surfaces, leading to rapid loss. Guo et al. analyzed 119 textile products and found that over 76% of QAC-treated textiles exhibited migration rates exceeding 50% after washing [127]. Simulated laundry tests released ΣQAC concentrations of 42.1 ng/mL for hand-washing and 22.4 ng/mL for machine-washing. These QACs caused dose-dependent immobilization in Daphnia magna, with 7% regions showing high ecological risk after wastewater treatment and 41% for direct discharge.

While the above general fabrication methods apply broadly, certain natural antimicrobial polymers—most notably chitosan—present intrinsic processability barriers that demand specific strategies beyond routine blending or finishing. Chitosan is notoriously difficult to process into high-quality fibers via conventional wet spinning or electrospinning due to its high solution viscosity, strong interchain electrostatic repulsion and poor solubility in common organic solvents [128,129]. To overcome these limitations, three specialized strategies have been developed and should be critically considered when designing chitosan-based antimicrobial fibers: (i) Chemical modification: Derivatization of chitosan’s amino or hydroxyl groups—such as carboxymethylation, quaternization (e.g., N-(2-hydroxypropyl)-3-trimethylammonium chitosan chloride), or thiolation—significantly improves solubility in neutral/alkaline aqueous solutions or polar organic solvents, reduces solution viscosity, and often preserves or even enhances antimicrobial activity due to permanent cationic charges [130]; (ii) Co-spinning with carrier polymers: A widely adopted practical approach involves blending chitosan with readily spinnable synthetic or natural polymers (e.g., poly(ethylene oxide), poly(vinyl alcohol), poly(ε-caprolactone), or silk fibroin) [48]. (iii) Advanced solvent systems and coaxial spinning: Ionic liquids (e.g., 1-ethyl-3-methylimidazolium acetate) and deep eutectic solvents can dissolve high-molecular-weight chitosan directly, enabling spinning without derivatization. Alternatively, coaxial electrospinning allows chitosan to be confined to the fiber surface (where antimicrobial action is needed) while a more spinnable polymer forms the core, decoupling processability from functionality [131].

In summary, graft modification provides optimal durability with minimal environmental impact, while finishing treatment, though economical, poses significant ecotoxicological risks. Future development should prioritize covalent grafting strategies to balance antimicrobial efficacy with environmental sustainability.

## 3. Conclusions and Outlook

It is important to note that the reported antimicrobial efficacy values (e.g., inhibition rates, log reduction) across different studies are not directly comparable due to the lack of universally adopted testing standards. Experimental conditions such as inoculum concentration (typically 10^5^–10^8^ CFU/mL), contact time (ranging from minutes to 24 h), static vs. dynamic incubation, bacterial strain variation (e.g., *E. coli* ATCC 25922 vs. other clinical isolates), and test methods (e.g., AATCC 100, ISO 20743, JIS L 1902 [132,133,134], or custom shake-flask methods) can significantly influence the measured outcomes. Therefore, in this review, the original experimental parameters were presented alongside the reported efficacies to provide context. Direct quantitative comparisons should be avoided unless identical testing protocols were employed.

In addition to performance and durability, the environmental safety of antimicrobial fibers warrants critical attention. Although this review focuses on antimicrobial efficacy and material durability, the potential ecotoxicological risks associated with the continuous release of antimicrobial agents—such as silver nanoparticles, copper and zinc ions, and synthetic N-halamines—during repeated washing cycles cannot be overlooked. These leached agents may accumulate in aquatic ecosystems, disrupt microbial communities, and contribute to long-term environmental pollution. Furthermore, antimicrobial synthetic fibers may shed microplastic particles during laundering and daily use, and these microplastics can act as vectors for leached biocides (e.g., Ag^+^, QACs, Cu^2+^), potentially increasing environmental toxicity and human exposure through inhalation or ingestion. Future research should therefore integrate life-cycle assessment, standardized washing simulation protocols, and ecotoxicity screening (e.g., using aquatic model organisms) to evaluate the environmental fate of these materials. Moreover, antimicrobial fiber design should prioritize covalent immobilization strategies that minimize agent leaching and consider biodegradability or closed-loop recycling to mitigate microplastic pollution. Addressing these environmental dimensions, including the development of intrinsically less harmful agents, biodegradable carriers, or effective capture systems for released actives, will be essential for the responsible translation of laboratory innovations into real-world applications.

Synthesizing the progress in antimicrobial fibers, this review has investigated both natural fibers with inherent bioactivity and engineered synthetic fibers modified through various strategies. A systematic analysis of antimicrobial agents, encompassing plant-derived extracts, inorganic nanoparticles, metal–organic frameworks (MOFs), and synthetic organic compounds, has clarified their distinct mechanisms, such as membrane disruption, metabolic interference, photocatalytic activity, and controlled ion release. Significantly, remarkable progress in durability and wash resistance has been achieved through advanced fabrication techniques like covalent grafting, microfluidic spinning, and composite design. The synergistic effects observed when integrating different antimicrobial systems improve performance and alleviate bacterial resistance. Collectively, these advancements offer a solid basis for high-performance antimicrobial textiles in medical, protective, and consumer applications. It is important to distinguish between the modification of natural cellulose-based fibers and synthetic polymers (e.g., polyethylene terephthalate (PET), polypropylene (PP), and polyamide (PA)). Synthetic polymers are typically hydrophobic and chemically inert, making the immobilization of antimicrobial agents more challenging compared to natural fibers. Strategies such as in situ polymerization and surface grafting (e.g., using plasma treatment or chemical cross-linkers) are often required to ensure durable attachment [77,86]. Addressing the durability issue is not only key to maintaining antimicrobial performance but also serves as a critical measure to prevent the leakage of biocides and the generation of contaminated microplastics during use.

Future research should prioritize the development of next-generation intelligent systems with stimuli-responsive activity (e.g., triggered by the presence of bacteria, pH variations, or temperature changes). Rechargeable biocidal platforms, especially regenerable N-halamine polymers, represent a promising approach for sustained efficacy with reduced resource consumption. There is significant potential in promoting green manufacturing and establishing sustainable material cycles, aligning production with the principles of the circular economy. The integration of nanotechnology, biotechnology, and materials science is likely to produce multifunctional fibers that combine antimicrobial properties with self-cleaning ability, environmental sensing, and wound-healing promotion. Moreover, establishing standardized evaluation protocols and conducting comprehensive toxicological assessments are essential for translating laboratory innovations into commercially viable products. As global healthcare and sustainability challenges change, antimicrobial fibers are set to be crucial components in innovative solutions for public health protection and advanced material applications.

## Figures and Tables

**Table 1:** Comparative summary of natural antimicrobial agents in fibrous materials: composition, efficacy determinants, processing effects, and dominant mechanisms.

Agent Source	Key Active Compounds	Typical Selectivity	Key Influence of Processing	Dominant Mechanism Under Realistic Conditions	Major Limitation for Fiber Application
Bamboo	Flavonoids (e.g., tricin), phenolics, stigmasterol	Moderate; often higher against Gram-negative (e.g., K. pneumoniae)	Mild alkali/thermal treatment removes nutrients and liberates insoluble phenolics, enhancing activity; strong delignification reduces efficacy	Membrane disruption by hydrophobic compounds; nutrient removal contributes to raw fibers	Low intrinsic potency; requires preservation of lignin-bound actives
Hemp (Cannabis)	Cannabinoids (e.g., CBD), flavonoids, phenolic acids	Broad-spectrum, including MRSA and some Gram-negative	Sensitive to heat, light, and organic solvents; encapsulation needed for durability	Membrane integration and metabolic interference; multi-target action lowers resistance risk	Volatility/degradation; poor wash fastness without protection
Chitosan	Protonated primary amines (-NH_3_^+^)	Broad-spectrum (both Gram+ and Gram−)	Strongly dependent on DD and Mw; wet-spinning and electrospinning preserve charge	Electrostatic disruption of anionic membranes (pH-dependent; optimal at acidic pH)	Activity drops at neutral/alkaline pH; mechanical properties limited
Jute	Phenolic acids, flavonoids, hydrophobic lignin	Broad-spectrum (Gram-positive and Gram-negative)	Chemical modifications (e.g., scouring, bleaching) can reduce or redistribute active components	Physical-chemical synergy: porous structure + phenolic membrane disruption	Inconsistent activity due to extraction method; fiber roughness can be a drawback
Essential oils (e.g., peppermint, cinnamon)	Terpenes, phenols (menthol, cinnamaldehyde, thymol)	Generally higher against Gram-positive; Gram-negative less susceptible due to LPS barrier	Highly volatile; requires encapsulation or ionic liquid spinning to retain activity; thermal processing degrades efficacy	Membrane fluidization and increased permeability; rapid contact killing	Poor durability and heat sensitivity; fast release leads to short lifetime
Other plant (sisal, coir)	Saponins, various phenolics	Variable; often moderate	pH-dependent extraction; limited systematic processing data	Membrane complexation (saponins) or general metabolic interference	Poorly characterized; low fiber-binding affinity

## Data Availability

No new data were created or analyzed in this study. Data sharing is not applicable to this article.
